# Differences in Pneumococcal and *Haemophilus influenzae* Natural Antibody Development in Papua New Guinean Children in the First Year of Life

**DOI:** 10.3389/fimmu.2021.725244

**Published:** 2021-08-10

**Authors:** Kelly M. Martinovich, Tasmina Rahman, Camilla de Gier, Elke J. Seppanen, Tilda Orami, Caitlyn M. Granland, Jacinta Francis, Mition Yoannes, Karli J. Corscadden, Rebecca Ford, Peter Jacoby, Anita H. J. van den Biggelaar, Lauren O. Bakaletz, Allan W. Cripps, Deborah Lehmann, Peter C. Richmond, William S. Pomat, Lea-Ann S. Kirkham, Ruth B. Thornton

**Affiliations:** ^1^Wesfarmers Centre of Vaccines and Infectious Diseases, Telethon Kids Institute, Perth, WA, Australia; ^2^Division of Paediatrics, University of Western Australia, Perth, WA, Australia; ^3^Papua New Guinea Institute of Medical Research, Goroka, Papua New Guinea; ^4^Centre for Child Health Research, University of Western Australia, Perth, WA, Australia; ^5^Center for Microbial Pathogenesis, Abigail Wexner Research Institute at Nationwide Children’s Hospital, The Ohio State University College of Medicine, Columbus, OH, United States; ^6^School of Medicine and Menzies Health Institute Queensland, Griffith University, Southport, QLD, Australia

**Keywords:** nontypeable *Haemophilus influenzae* (NTHi), pneumococcus, protein IgG, natural antibody, Papua New Guinea, vaccines

## Abstract

**Background:**

Development of vaccines to prevent disease and death from *Streptococcus pneumoniae*, and nontypeable *Haemophilus influenzae* (NTHi), the main pathogens that cause otitis media, pneumonia, meningitis and sepsis, are a global priority. Children living in low and lower-middle income settings are at the highest risk of contracting and dying from these diseases. Improved vaccines with broader coverage are required. Data on the natural development of antibodies to putative vaccine antigens, especially in high-risk settings, can inform the rational selection of the best antigens for vaccine development.

**Methods:**

Serum IgG titres to four pneumococcal proteins (PspA1, PspA2, CbpA, and Ply) and five NTHi antigens (P4, P6, OMP26, rsPilA and ChimV4) were measured in sera collected from 101 Papua New Guinean children at 1, 4, 9, 10, 23 and 24 months of age using multiplexed bead-based immunoassays. Carriage density of *S. pneumoniae* and *H. influenzae* were assessed by quantitative PCR on genomic DNA extracted from nasopharyngeal swabs using species-specific primers and probes. All data were log-transformed for analysis using Student’s unpaired t-tests with geometric mean titre (GMT) or density (GMD) calculated with 95% confidence intervals (CI).

**Results:**

Serum -pneumococcal protein-specific IgG titres followed a “U” shaped pattern, with a decrease in presumably maternally-derived IgG titres between 1 and 4 months of age and returning to similar levels as those measured at 1 month of age by 24 months of age. In contrast, NTHi protein-specific IgG titres steadily increased with age. There was no correlation between antibody titres and carriage density for either pathogen.

**Conclusion:**

This longitudinal study indicates that the waning of maternally- derived antibodies that is usually observed in infants, after infants does not occur for NTHi antigens in Papua New Guinean infants. Whether NTHi antigen IgG can be transferred maternally remains to be determined. Vaccines that are designed to specifically increase the presence of protective NTHi antibodies in the first few months of life may be most effective in reducing NTHi disease.

**Clinical Trial Registration:**

https://clinicaltrials.gov/, identifier NCT01619462.

## Highlights

1. Pneumococcal protein IgG levels followed traditional trajectories early in life.
2. Haemophilus protein IgG levels did not, increasing from 1 to 24 months of age.
3. Early and high carriage density was observed irrespective of protein IgG titres.

## Introduction

*Streptococcus pneumoniae* and *Haemophilus influenzae* infections are a major cause of infant morbidity and mortality and the development of otitis media (OM) pneumonia, meningitis and sepsis (1, 2), particularly in low- and middle-income countries (3). Protein-based vaccines are being developed against *S. pneumoniae* and *H. influenzae* to provide broader protection than the existing *S. pneumoniae* serotype-specific polysaccharide vaccines (4). Protein-based vaccines may overcome serotype replacement issues that have emerged following the introduction of pneumococcal conjugate vaccines (PCVs) (5). Adding additional serotypes into these vaccines can overcome serotype replacement in the short term but, with over 100 pneumococcal serotypes, formulation of a serotype-based pneumococcal vaccine is not possible due to cost and antigen volume required (6, 7).

Proteins have been successfully used to enhance the T-cell dependant immune response of pneumococcal and *H. influenzae* polysaccharide vaccines including PCVs and the *H. influenzae type B* vaccine (8). The non-typeable *Haemophilus influenzae* (NTHi) outer membrane protein, Protein D (PD), has been successfully used as a conjugate protein to induce T-cell dependent immune memory to pneumococcal polysaccharides included in the 10-valent PCV. PD has also been trialled as a sub-unit vaccine antigen for NTHi, with immunogenicity and safety demonstrated in infants (9) and adults (10). PD immunisation in a mouse model of OM showed reduced NTHi loads in the middle ear but not the lung (11). In addition to NTHi PD, several other conserved NTHi surface antigens are being, or have been, assessed for vaccine development including recombinant soluble type IV pilus majority sub-unit A protein (rsPilA), outer membrane protein 5 (P5) (12), outer membrane protein 26 (OMP26) (13), Protein E (PE), outer membrane protein 4 (P4) and outer membrane protein 6 (P6) (14). Additionally, a fusion protein called ChimV4 that contains rsPilA and P5 epitopes and has been shown to be protective against NTHi-induced OM in a chinchilla model (14). A tri-valent protein vaccine containing PD, a fusion protein containing both PE and rsPilA epitopes and ubiquitous surface protein A2 (UspA2) protein from *Moraxella catarrhalis* was immunogenic and safe in adults (15). In a Phase II clinical trial the tri-valent vaccine given in a 2-dose schedule had limited efficacy (13.3%) against acute exacerbations in adults with chronic obstructive pulmonary disease (16) and is now being investigated using a 3-dose schedule (NCT03443427).

Pneumococcal protein vaccines have been under development for decades, with demonstrated immunogenicity of polyhistidine triad Protein D (PhtD) and choline-binding protein A (CbpA) in Phase I clinical trials in both monovalent and bivalent formulations (17, 18). Detoxified pneumolysin (dPly) and PhtD, either alone or combined with PCV have also been proven to be safe and immunogenic in children from The Gambia (10, 19, 20), but efficacy data are lacking. The dPly/PhtD protein vaccine was recently compared to PCV13 in a Phase 2b trial in Native American infants and was proven safe and immunogenic but failed to improve the prevention of acute OM when compared with PCV13 (21). Two multi-antigen pneumococcal vaccines have also been investigated, one containing the recombinant proteins CbpA, PhtD, and dPly (22) and another containing dPly, pneumococcal surface adhesin A (PsaA), Pneumococcal Iron Uptake Protein A (PiuA) and pneumococcal surface protein A family 2 (PspA2) antigens (PnuBioVax™). These vaccines have both been shown to be safe and immunogenic in Phase I trials (23). Fusion proteins containing PsaA and PspA epitopes (24) and CbpA and dPly-with L460D substitution (25) are being developed as vaccine candidates (24). Combinations of full-length or fragments of dPly, CbpA, PspA and dPly-with L460D substitution are also being investigated (25, 26).

Understanding the natural development of antibodies against candidate protein vaccine antigens in different populations helps to assess where and when protein vaccines may be most useful. In addition, establishing a correlate of protection for pneumococcal or NTHi protein-specific antibodies against disease or colonisation would support and accelerate vaccine development. Nasopharyngeal colonisation with *S. pneumoniae* or *H. influenzae* is the precursor for development of pneumococcal or *H. influenzae* disease and mediates host-to-host transmission (27, 28). In areas with high rates of pneumococcal and *H. influenzae* disease, colonisation occurs within weeks of birth. For example, infants in Papua New Guinea (PNG) have a median age of acquisition of *S. pneumoniae* of 19 days old (29), with ~80% of children colonised with *S. pneumoniae* by 3 months of age (30) and over 90% colonised with NTHi by 4 months of age (31). Vaccines to prevent or reduce density of colonisation are likely to have the greatest impact on decreasing morbidity and mortality.

The aim of this study was to demonstrate natural antibody development against putative protein vaccine antigens in Papua New Guinean infants who experience one of the highest rates of respiratory infections in the world. Naturally acquired IgG titres to pneumococcal (PspA1, PspA2, CbpA and Ply) and NTHi antigens (P4, P6, OMP26, rsPilA and ChimV4) were measured in serum collected from children over the first two years of life. Correlations between antibody titres and colonisation density were assessed to determine if protein vaccines might be used to redirect or boost antibody to protect against disease.

## Methods

### Study Cohort

Serum and nasopharyngeal swabs were collected from children at 1, 4, 9, 10, 23 and 24 months of age as part of a clinical trial assessing the safety and immunogenicity of the 10-valent and 13-valent PCVs and a 23-valent pneumococcal polysaccharide vaccine (PPV23) booster at age 9 months in Papua New Guinean infants (32). For this study, children who had both serum and nasopharyngeal swabs collected for at least 5 of the 6 visits were included (n=101) as previously described (31). The study was conducted according to Declaration of Helsinki International Conference on Harmonisation Good Clinical Practice (ICH-GCP). Ethics approval was obtained from the PNG Medical Research Advisory Committee (#11.03), Papua New Guinea Institute of Medical Research (PNGIMR) Institutional Review Board (#1028) and University of Western Australia Human Research Ethics Committee (Approval # RA/4/1/7309). Full details of the consent process have been described elsewhere (32). The study was registered with ClinicalTrials.gov CTN NCT01619462.

### Sample Collection and Processing

Venous blood samples were collected from study participants, followed by serum separation and aliquoting at the PNGIMR in Goroka. Sera were stored at -80°C. Nasopharyngeal swabs were collected and placed in 1mL of skim-milk tryptone-glucose-glycerol broth as previously described (33, 34), and stored at −80°C within 2 hours of collection. Specimens were then cryogenically shipped from PNGIMR to the Telethon Kids Institute, Western Australia for antibody and carriage density measurements.

### Detection of *S. pneumoniae* and *H. influenzae* Carriage Density Using qPCR

The swab media was enzymatically lysed and genomic DNA (gDNA) extracted as previously described (35). Quantitative (q)PCR was conducted using primers and probes specific for *S. pneumoniae: lytA* (36), and *H. influenzae: fuc*P and *hpd*3 as described previously (37, 38). Analysis of the qPCR data was conducted using the Bio-rad CFX manager 3.1 (Bio-Rad) and log-transformed and graphed as pg/mL.

### Measurement of IgG to Pneumococcal and NTHi Antigens by Multiplex Bead-Based Assay

Sera were assessed for protein specific IgG against the pneumococcal proteins PspA1, PspA2, CbpA, Ply (ST306) and NTHi antigens PD, P4, P6 using 2 multiplexed bead-based immunoassays described previously (39). Three additional *H. influenzae* antigens, OMP26, rsPilA and ChimV4, were included in these multiplex assays following inhibition and cross-reactivity studies as previously described (39). The final assays consisted of a 5-plex immunoassay containing PspA1, PD, ScpA (*Streptococcus pyogenes* antigen, data not shown here), OMP26 and rsPilA, and a 6-plex immunoassay containing PspA2, CbpA, Ply, P4, P6 and ChimV4. Serum samples were diluted 1:100 for the 5-plex and 1:300 for the 6-plex in sample diluent (phosphate buffered saline containing 2% newborn bovine serum and 0.05% Tween 20 - both from Sigma-Aldrich). A 3-fold dilution of standards was prepared at a starting ratio of 1:20 for the 5-plex assay and 1:200 for the 6-plex assay. Diluted serum (25µL) and standards (25µL) were mixed with prepared beads and incubated in the dark at room temperature for 30 minutes followed by the standard bioplex protocol (39). The mean fluorescence intensity (MFI) values were generated with the Bio-plex Manager 6.0 software and calculated as arbitrary units (AU/mL). Inter-assay variability was assessed using the % of coefficient of variation (CV) of MFI of the standard curve and remained within 10-15% between plates. Out of range readings were repeated at an appropriate higher or lower dilution to fit within the standard curve.

### Statistical Analyses

All data were non-parametric and therefore log-transformed to normalise prior to analysis using Student’s unpaired t-tests. Geometric mean titre (GMT) or density (GMD) was calculated with 95% confidence intervals (CI). Linear regression was used to assess potential relationships between pneumococcal density or NTHi density and IgG titres. p ≤ 0.05 was considered significant. All data were analysed using SPSS version 25 (IBM, Armonk, New York, United States of America) and graphs were prepared with GraphPad Prism 8 (GraphPad, La Jolla, California, United States of America).

## Results

### Study Cohort

Of the 101 children included in this analysis, 55 had received PCV10 (32 (58%) Female) and 46 PCV13 (23 (50%) Female) as part of the vaccine trial at 1, 2, and 3 months of age. The proportion of children in each PCV group who received a dose of the 23-valent pneumococcal polysaccharide vaccine at 9 months of age was similar (33; 60.0%) for PCV10-vaccinated children and PCV13-vaccinated children (27; 58.7%) (p=0.894).

The GMTs of serum IgG to all pneumococcal and NTHi antigens were comparable between the PCV10- and PCV13-vaccinated groups at all time points (**Supplementary Table 1**), except for anti-CbpA IgG which was significantly lower in the PCV13-vaccinated group at 10 months of age. We therefore combined responses for the PCV10- and PCV13-vaccinated groups for longitudinal analysis of natural IgG responses to protein antigens. When analyses were broken down according to those children receiving PPV23 at 9 months compared to those who had not, an increase in P4-specific IgG levels was observed at 24 months of age for children vaccinated with PCV10+PPV23 compared to those not receiving the booster (p=0.015) (**Supplementary Table 2**). As this was the only significant difference and unlikely to be explained biologically, analyses were performed for PPV recipients and non-recipients as one group.

### Natural Serum IgG Titres to Pneumococcal Proteins Waned in the First Months of Life and Returned Close to Baseline by 2 Years of Age

**Figure 1:**
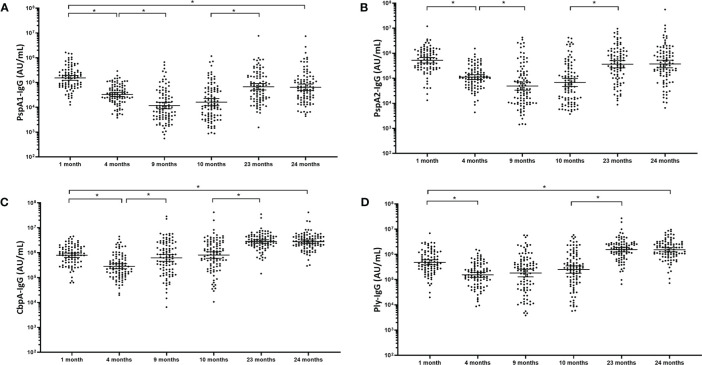
Serum IgG antibodies against pneumococcal proteins waned in the first months of life and returned close to baseline by 2 years of age. Each point represents the serum IgG titres against pneumococcal proteins for a child in AU/mL **(A)** PspA1, **(B)** PspA2, **(C)** CbpA and **(D)** Ply in children from PNG at different ages. The horizontal bars depict the geometric mean titre (GMT) of IgG at each time-point +/- 95% CI. PspA1, pneumococcal surface protein A family 1; PspA2, pneumococcal surface protein A family 2; CbpA, choline-binding protein A; Ply, pneumolysin. *p-value <0.05, statistical analyses were conducted on the logarithmically transformed data. All children had measurable antibody titres.

Antibody titres against all four pneumococcal proteins followed the traditional “U-shaped” curve over the first 2 years of life. IgG titres to all four pneumococcal proteins were significantly lower at 4 and 9 months of age in comparison with the 1-month titres (**Figure 1**). For PspA1 and PspA2 (**Figures 1A, B**), titres were 4-fold lower at 4 months of age compared to 1 month of age (p<0.001). Titres continued to decline by 3-fold at 9 months of age in comparison with 4-month titres (p<0.001), before increasing 4.3-fold (PspA1) and 5.6-fold (PspA2) between 10 months and 23 months of age (p<0.001). CbpA-IgG titres (**Figure 1C**) decreased 2.8-fold from 1 month to 4 months of age (p<0.001) before increasing 2-fold at 9 months of age (p<0.001). The titres increased 3.7-fold from 10 months to 23 months. The pattern of Ply-specific IgG (**Figure 1D**) was similar to the other pneumococcal proteins, showing a 3-fold decrease from 1 month to 4 months of age (p<0.001) and remaining constant at 9 and 10 months of age (p=0.200). Ply-IgG titres then increased 6.7-fold from 10 to 23 months of age (p<0.001). No changes were observed in any anti-pneumococcal protein titres between 23 and 24months of age (P=0.827). CbpA- and Ply-specific IgG titres were 3-fold higher at 24 months compared to 1 month (p<0.001) whereas PspA1 and PspA2 were 2.5 (p<0.001) and 1.5-fold (p=0.116) lower respectively at 24 months compared to titres at 1 month of age.

### Naturally Acquired Serum IgG Titres to NTHi Antigens Increase in the First 2 Years of Life

**Figure 2:**
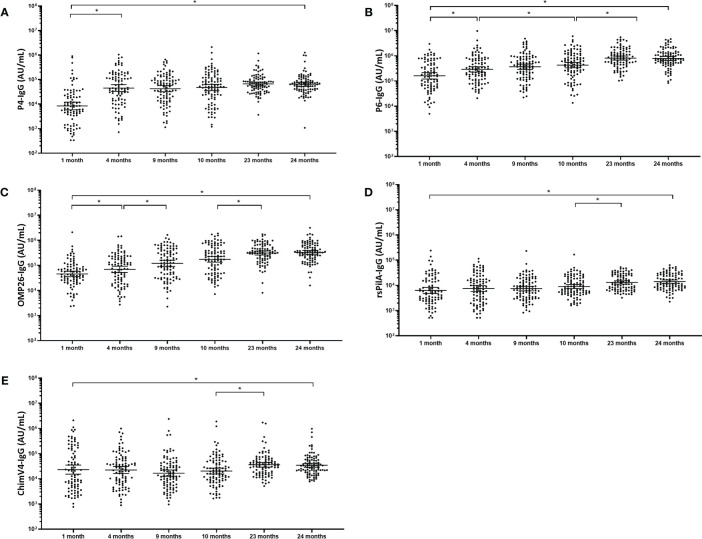
Serum IgG antibodies against NTHi antigens increase gradually over the first 2 years of life. Each point represents the serum IgG titre against NTHi antigens for a child in AU/mL **(A)** P4, **(B)** P6, **(C)** OMP26 and **(D)** rsPilA **(E)** ChimV4. The horizontal bars depict the geometric mean titre (GMT) of IgG at each time-point +/- 95% CI. PD, Protein D; P4, Protein 4; P6, outer membrane protein 6, OMP26, outer membrane protein 26; rsPilA, recombinant soluble pilus A protein; ChimV4, chimeric vaccine antigen 4 (rsPilA and P5). *p-value <0.05, Statistical analyses were conducted logarithmically transformed data. All children had measurable antibody titres.

In contrast to pneumococcal protein-specific IgG, no “U-shaped” curves were observed for antibodies developed against NTHi antigens over the first two years of life. From 1 month to 4 months of age serum IgG titres for P4 (5.6-fold; p<0.001), P6 (1.8-fold; p<0.01) and OMP26 (1.5-fold; p<0.001) significantly increased, while for rsPilA (1.2-fold; p=0.383) and ChimV4 (1-fold; p=0.887) IgG titres did not change. P4-specific IgG titres increased from 4 months to 24 months although this was not significant (1.6-fold increase; p=0.058). A 1.5-fold increase between 4 and 10 months of age was observed for P6-specific IgG (p=0.030), increasing a further 2.2-fold by 23 months of age (p<0.001). OMP26-specific IgG increased 1.7-fold between 4 months and 9 months of age (p=0.007) and 1.9-fold from 10 to 23 months of age OMP26 (p<0.001). Delayed antibody development to both PilA and ChimV4 was observed, with titres increasing 1.5-fold and 1.8-fold respectively between 10 and 23 months of age (p<0.001). All NTHi IgG titres remained steady between 23 and 24 months of age. All natural NTHi antigen IgG titres were higher at 24 months compared to 1 month of age (p<0.001), (**Figure 2**) with P4 increasing 8.7-fold (p<0.001), P6 5.2-fold (p<0.001), OMP26 7.6-fold (p<0.001), rsPilA 2.4-fold (p<0.001) and ChimV4 1.7-fold (p=0.076). PD-specific IgG titres have been published elsewhere for this cohort (31).

### Pathogen Specific Antibody Titres Do Not Correlate With *S. pneumoniae* and *H. influenzae* Carriage Density in the First Two Years of Life

Seventy percent of the cohort were colonised with the pneumococcus by 1 month of age, increasing to 93% by 2 years of age. Of the children that were colonised with *S. pneumoniae*, carriage density was constant for the first 10 months of age [GMD = 14,260 pg/mL (95% CI 8459-24,028 pg/mL; 1 month), 15,107 pg/mL (95% CI 9584-23,812 pg/mL; 4 months), 14,167 pg/mL (95% CI 9393-21,369 pg/mL; 9 months) and 15,175 pg/mL (95% CI 10,127-22,739 pg/mL; 10 months) (**Supplementary Figure 1**)]. There was a significant increase in pneumococcal carriage density between 10 and 23 months of age (29,048 pg/mL, 95% CI 19,392-43,512 pg/mL; 23 months) (p=0.025), before decreasing non-significantly at 24 months to 23,908 pg/mL (95% CI of 15,279-37,412 pg/mL; 24 months) (p=0.137); this may be vaccine related and investigation is ongoing. There were no correlations between pneumococcal carriage density and serum IgG titres to any of the tested pneumococcal proteins at any of the time points (p>0.104) (**Supplementary Table 3**). For *H. influenzae*, ~52% of children were colonised at 1 month of age, increasing to 100% by 2 years of age (31). Of the children that were colonised with *H. influenzae*, carriage density was constant for the first 10 months of age [GMD= 12,126pg/mL (95% CI 5601-26,252 pg/mL; 1 month) (20,280 pg/mL; 95% CI 11,787-34,895 pg/mL; 4 months) 16,301 pg/mL (95% CI 9601-27,675 pg/mL 9 months) 9532 pg/mL (95% CI 5833-15,577 pg/mL 10 months) **Supplementary Figure 2**]. There was a significant increase in *H. influenzae* carriage density between 10 and 23 months of age 23,084 pg/mL (95% CI 14,062-37,895 pg/mL 23 months; p=0.014) before decreasing non-significantly at 24 months to 18,773 pg/mL (95% CI 10,884-32,382 pg/mL 24 months, p=0.793) *H. influenzae* density did not correlate with *H. influenzae* antibody titres at any of the time points (p>0.077) (**Supplementary Table 4**). No assessment of antibody data based on presence or absence of either pathogen could be conducted due to the extremely high rates of carriage in this cohort resulting in numbers of non-carriers being too low for meaningful analysis.

## Discussion

We have demonstrated substantial differences in the patterns of antibody acquisition and development to NTHi and pneumococcal antigens in the first 2 years of life despite similar levels of colonisation. Pneumococcal antibody followed the traditional “U-shaped” curve indicative of waning maternal antibody prior to development of the infant’s own antibody in response to pathogen exposure. In contrast to this, NTHi antibody titres were low early in life with no obvious transfer and subsequent waning of maternal antibody, rather only a gradual increase over time in response to repeated carriage events. Our previous studies assessing the development of natural immunity to the same pneumococcal proteins in serum from Australian children from 6 months to 14 years of age also demonstrated that antibody titres increase with age (39, 40). Longitudinal studies of younger children in PNG and the Philippines have also observed waning of maternal-derived pneumococcal antibodies in the first months of life, with serum PspA1 and PspA2 antibody titres waning in PNG children before increasing between 9 and 18 months of age (41). Similarly, in the Filipino study, maternal antibody levels declined over the first 18 weeks of life before an increase in self-produced antibody was observed (42). These rises in pneumococcal antibody were associated with pneumococcal carriage acquisition in both studies (41, 43). Differences in the timing of waning immunity was observed for the different pneumococcal proteins, with antibodies to CbpA and Ply at the lowest titre at 4 months of age compared with PspA1/2 antibody titres that were at their lowest at 9 months of age. It is possible that this may be due to the incidence of exposure to these antigens, and perhaps maternal antibodies to the ubiquitous CbpA and Ply are depleted more quickly than to PspA due to the heterogeneity within the PspA family. However, insight into the molecular epidemiology of the circulating pneumococci is required to investigate this further. Antibodies from humans vaccinated with fragments of recombinant PspA have been demonstrated to offer cross-protection from invasive disease with S. pneumoniae expressing heterologous PspA when administered to mice (44).

There are limited reports on the development of antibodies to NTHi antigens in young children. A Chinese cross-sectional study of hospital attendees aged 0-99 years old found a similar increase in P6 (and PD) titres in the first 2 years of life as observed in our study (45). In Australian aboriginal children a gradual increase in NTHi antigen antibodies P4, P6 and PD was also observed from 6 months to 14 years of age (46). Interestingly, anti-NTHi IgG titres were lowest in adults aged 20-40 years old in the Chinese cohort, corresponding to child-bearing age and this may explain the lack of maternally-transferred antibody observed in our study. Previously, P6 and OMP26-specific serum IgG titres have been reported to increase between 6 and 30 months of age in North American children and further increased (for P6 only) if children were colonised with NTHi (47). In this study, we could not disaggregate responses based on NTHi colonisation status as the majority of children were colonised by 4 months of age; however, the gradual increase of NTHi antibodies in the first 2 years of life in PNG children is in line with findings for other paediatric populations (45, 47).

Our finding that IgG levels to NTHi antigens in PNG infants did not decline in the first months of life and only increased with age, suggests that infants do not receive maternally-derived NTHi protein-specific antibodies. There are two plausible explanations for this: 1) that the NTHi antibodies do not cross the placenta or 2) that mothers (or indeed women of child-bearing age) do not have NTHi antibodies to transfer. There are data to support preferential transfer of antibodies based on their functional potential in neonates, examples of which include specific antigens against pertussis and poliovirus (48, 49). The second theory is supported by the Hua et al. (45) Chinese study, where IgG titres to NTHi PD and P6 were lowest in the child-bearing years (45). While we did not measure maternal antibody titres in this study, future studies assessing maternal, cord blood and breast milk NTHi IgA and IgG antibody levels are warranted in different populations. This may be related to adults being less likely to carry NTHi than children and thus not being triggered to produce antibody at a titre sufficient to transfer across the placenta, however this requires further investigation. It does however suggest that boosting titres of protective NTHi antibodies that promote bacterial clearance in women of child-bearing age through maternal vaccination strategies could be the most effective way to protect from NTHi disease early in life.

We have demonstrated that an early and high density of *S. pneumoniae* and *H. influenzae* colonisation elicits development of antigen-specific antibodies in PNG infants, however, these do not reduce further pathogen acquisition and are unlikely to reduce disease given the high rates of acute lower respiratory infection observed in this cohort (34). While sterilising immunity may not be a desirable outcome for vaccines against respiratory pathogens, reducing colonisation density is important for reducing transmission, and thus reducing the disease burden. Antibodies to different epitopes of the same antigen may improve the ability to redirect the immune system towards pathogen clearance or protection from opportunistic infection (12). Epitope mapping of the immunodominant regions of rsPilA, and subsequent design of the chimeric antigen, ChimV4, has revealed that antibodies directed against the C-terminal rsPilA epitope were critical for protection from NTHi disease, but antibodies directed against the N-terminal of rsPilA were not (12). Chimeras of NTHi DNABII proteins were also immunogenic against NTHi biofilm *in vitro* (50). Likewise, a PD peptide was shown to elicit a superior immunogenic response compared to the whole PD protein (51). These mapping studies should also be performed for pneumococcal antigens to further understand vaccine potential and design.

In summary, we have demonstrated that Papua New Guinean children naturally develop IgG antibodies to pneumococcal and NTHi antigens within the first years of life. The pattern of antibody development varied for pneumococcal and NTHi antigens, with the suggestion that less maternal antibodies for NTHi antigens are passed to infant. No correlation was observed between antibody titres and carriage density for either pathogen. Maternal delivery of carefully designed NTHi protein vaccines may be suitable approach to provide early-in-life protection against NTHi-associated disease, especially in high-risk populations.

## Data Availability Statement

The original contributions presented in the study are included in the article/**Supplementary Material**. Further inquiries can be directed to the corresponding author.

## Ethics Statement

The studies involving human participants were reviewed and approved by PNG Medical Research Advisory Committee. Written informed consent to participate in this study was provided by the participants’ legal guardian/next of kin.

## Author Contributions

TR and KC conducted the serology testing on the sera. KM and TR analysed the antibody data. CdG developed the qPCR on the nasopharyngeal swabs and conducted and analysed the qPCR data together with CMG. ES and KM merged the antibody and carriage datasets, analysed the compiled data, and contributed to manuscript writing and data interpretation. JF and RF coordinated and conducted sample collection and processing as well as shipment of specimens from Goroka to Perth. PJ assisted with statistical analyses. DL, WP, RT, and L-AK oversaw experimental conduct and analyses. AB, DL, PR, WP, RT, and L-AK contributed to data interpretation. All authors contributed to the article and approved the submitted version.

## Funding

This work was supported by funding from the Wesfarmers Centre of Vaccines and Infectious Diseases, Telethon Kids Institute and the Australian National Health and Medical Research Council (NHMRC) project #1087200. L-AK was supported by an NHMRC Career Development Fellowship #1061428 during this project.

## Conflict of Interest

WP has received funding from Pfizer Australia to attend a conference. AB conducts part-time consultancy work for vaccine companies on projects not related to this study. L-AK has received investigator-initiated research grants, educational grants and travel support from Pfizer, GlaxoSmithKline and Merck, Sharp & Dohme, and is an inventor on patents for a pneumococcal protein vaccine antigen. DL is an investigator on an investigator-initiated research grant that was funded by Pfizer Australia. PR has received nonfinancial support from Pfizer, grants from GlaxoSmithKline and Pfizer, and nonfinancial support from GlaxoSmithKline for work outside the submitted work. The Papua New Guinea Institute of Medical Research received sponsorship from Pfizer Australia to host a national Medical Symposium in 2014. AC is a member of the Seqirus Australia Pneumococcal Advisory Board and the Merck & Co Global Pneumococcal Advisory Board. LB has received grants from GlaxoSmithKline to develop NTHi type IV pilus-derived vaccine antigens.

The remaining authors declare that the research was conducted in the absence of any commercial or financial relationships that could be construed as a potential conflict of interest.

## Publisher’s Note

All claims expressed in this article are solely those of the authors and do not necessarily represent those of their affiliated organizations, or those of the publisher, the editors and the reviewers. Any product that may be evaluated in this article, or claim that may be made by its manufacturer, is not guaranteed or endorsed by the publisher.

## Supplementary Material

Supplementary Table 1 Nontypeable *Haemophilus influenzae* and *Streptococcus pneumoniae* antigen serum IgG GMTs at each age after either PCV10 and PCV13 vaccinated groups. As we included children who had only 5 of the 6 serum samples collected in this analysis some time points do not have n=101. All values are AU/mL.

Supplementary Table 2 Nontypeable *Haemophilus influenzae* and *Streptococcus pneumoniae* antigen serum IgG GMTs compared between PPV23 recipients and non-recipients for those who received PCV10 or PCV13. As we included children who had only 5 of the 6 serum samples collected in this analysis some time points do not have n=101. All values are AU/mL.

Supplementary Table 3 *S. pneumoniae* density correlated with *S. pneumoniae* protein-specific IgG titre at different age time points. As we included children who had only 5 of the 6 serum samples collected in this analysis, time points do not have n=101.

Supplementary Table 4 NTHi density in swabs correlated with NTHi antigen titres. As we included children who had only 5 of the 6 serum samples collected in this analysis, time points do not have n=101.

Supplementary Figure 1 *S. pneumoniae* density in children carrying *S. pneumoniae* up to 2 years of age. *S. pneumoniae* nasopharyngeal density in swabs from children taken at 1, 4, 9, 10, 23 and 24 months of age. Data are presented for children that were colonised with *S. pneumoniae* as determined by qPCR. Each point represents an individual child, and the horizontal bars depict the median geometric mean density of DNA concentration in µg/mL in qPCR-positive samples. The number and percentages of children who were positive for *S. pneumoniae* carriage (*S. pneumoniae* DNA detected above the assay limit of detection) is outlined underneath the graph. *p-value < 0.05.

Supplementary Figure 2 NTHi density in children carrying NTHi up to 2 years of age. NTHi nasopharyngeal density in swabs from children taken at 1, 4, 9, 10, 23 and 24 months of age. Data are presented for children that were colonised with NTHi as determined by qPCR. Each point represents an individual child, and the horizontal bars depict the median geometric mean density of DNA concentration in µg/mL in qPCR-positive samples. The number and percentages of children who were positive for NTHi carriage (NTHi DNA detected above the assay limit of detection) is outlined underneath the graph. *p-value < 0.05.

## Appendix

The 10v13vPCV Trial Team (investigators in alphabetical order): Papua New Guinea Institute of Medical Research: L. Bele, M Dreyam, A. Elizah, R. Ford, J. Francis, A. Gihigupa, A. Greenhill, S. Javati, J. Kave, W. Kirarock, M. Lai, B. Martin, G. Masiria, A. Michael (deceased), L. Moliki, B. Nagepu, M. Nenikuro, B. Nivio, C. Opa, T. Orami, WS. Pomat, G. Saleu, P. Siba, V. Solomon, S. Wana, L. Wawae, M. Yoannes. Eastern Highlands Provincial Hospital: I. Hwaihwanje, T. Korowi, C. Mond, P. Wari. Telethon Kids Institute: A. van den Biggelaar, K. Corscadden, P. Jacoby, D. Lehmann. University of Western Australia: C. de Gier, L. Kirkham, T. Rahman, P. Richmond, R. Thornton. University of Sydney: M. Passey.

## References

[1] Lynch JP 3rd Zhanel GG. Streptococcus Pneumoniae: Epidemiology, Risk Factors, and Strategies for Prevention. Semin Respir Crit Care Med (2009) 30(2):189–209. 10.1055/s-0029-1202938 19296419

[2] Slack MPE. The Evidence for Non-Typeable Haemophilus Influenzae as a Causative Agent of Childhood Pneumonia. Pneumonia (Nathan) (2017) 9:9. 10.1186/s41479-017-0033-2 28702311 PMC5483294

[3] Bryce J Boschi-Pinto C Shibuya K Black RE. WHO Estimates of the Causes of Death in Children. Lancet (2005) 365(9465):1147–52. 10.1016/S0140-6736(05)71877-8 15794969

[4] Lagousi T Basdeki P Routsias J Spoulou V. Novel Protein-Based Pneumococcal Vaccines: Assessing the Use of Distinct Protein Fragments Instead of Full-Length Proteins as Vaccine Antigens. Vaccines (Basel) (2019) 7(1):9. 10.3390/vaccines7010009 PMC6466302 30669439

[5] Lewnard JA Hanage WP. Making Sense of Differences in Pneumococcal Serotype Replacement. Lancet Infect Dis (2019) 19(6):e213–20. 10.1016/S1473-3099(18)30660-1 30709666

[6] Løchen A Croucher NJ Anderson RM. Divergent Serotype Replacement Trends and Increasing Diversity in Pneumococcal Disease in High Income Settings Reduce the Benefit of Expanding Vaccine Valency. Sci Rep (2020) 10(1):18977. 10.1038/s41598-020-75691-5 33149149 PMC7643077

[7] Lee C Choi SK Kim RK Kim H Whang YH Pharm H. Development of a New 15-Valent Pneumococcal Conjugate Vaccine (PCV15) and Evaluation of Its Immunogenicity. Biologicals (2019) 61:32–7. 10.1016/j.biologicals.2019.07.005 31416790

[8] Pichichero ME. Protein Carriers of Conjugate Vaccines: Characteristics, Development, and Clinical Trials. Hum Vaccines Immunother (2013) 9(12):2505–23. 10.4161/hv.26109 PMC4162048 23955057

[9] Prymula R Pazdiora P Traskine M Rüggeberg JU Borys D. Safety and Immunogenicity of an Investigational Vaccine Containing Two Common Pneumococcal Proteins in Toddlers: A Phase II Randomized Clinical Trial. Vaccine (2014) 32(25):3025–34. 10.1016/j.vaccine.2014.03.066 24699466

[10] Leroux-Roels G Maes C De Boever F Traskine M Rüggeberg JU Borys D. Safety, Reactogenicity and Immunogenicity of a Novel Pneumococcal Protein-Based Vaccine in Adults: A Phase I/II Randomized Clinical Study. Vaccine (2014) 32(50):6838–46. 10.1016/j.vaccine.2014.02.052 24607003

[11] Michel LV Kaur R Zavorin M Pryharski K Khan MN LaClair C. Intranasal Coinfection Model Allows for Assessment of Protein Vaccines Against Nontypeable Haemophilus Influenzae in Mice. J Med Microbiol (2018) 67(10):1527–32. 10.1099/jmm.0.000827 30136923

[12] Novotny LA Adams LD Kang DR Wiet GJ Cai X Sethi S. Epitope Mapping Immunodominant Regions of the PilA Protein of Nontypeable Haemophilus Influenzae (NTHI) to Facilitate the Design of Two Novel Chimeric Vaccine Candidates. Vaccine (2009) 28(1):279–89. 10.1016/j.vaccine.2009.08.017 PMC2787809 19699813

[13] Kyd JM Cripps AW Novotny LA Bakaletz LO. Efficacy of the 26-Kilodalton Outer Membrane Protein and Two P5 Fimbrin-Derived Immunogens to Induce Clearance of Nontypeable Haemophilus Influenzae From the Rat Middle Ear and Lungs as Well as From the Chinchilla Middle Ear and Nasopharynx. Infect Immun (2003) 71(8):4691–9. 10.1128/IAI.71.8.4691-4699.2003 PMC165997 12874350

[14] Ysebaert C Denoël P Weynants V Bakaletz LO Novotny LA Godfroid F. A Protein E-PilA Fusion Protein Shows Vaccine Potential Against Nontypeable Haemophilus Influenzae in Mice and Chinchillas. Infect Immun (2019) 87(8):e00345–19. 10.1128/IAI.00345-19 PMC6652774 31109946

[15] Van Damme P Leroux-Roels G Vandermeulen C De Ryck I Tasciotti A Dozot M. Safety and Immunogenicity of non-Typeable Haemophilus Influenzae-Moraxella Catarrhalis Vaccine. Vaccine (2019) 37(23):3113–22. 10.1016/j.vaccine.2019.04.041 31029515

[16] Wilkinson TMA Schembri S Brightling C Bakerly ND Lewis K MacNee W. Non-Typeable Haemophilus Influenzae Protein Vaccine in Adults With COPD: A Phase 2 Clinical Trial. Vaccine (2019) 37(41):6102–11. 10.1016/j.vaccine.2019.07.100 31447126

[17] Seiberling M Bologa M Brookes R Ochs M Go K Neveu D. Safety and Immunogenicity of a Pneumococcal Histidine Triad Protein D Vaccine Candidate in Adults. Vaccine (2012) 30(52):7455–60. 10.1016/j.vaccine.2012.10.080 23131206

[18] Bologa M Kamtchoua T Hopfer R Sheng X Hicks B Bixler G. Safety and Immunogenicity of Pneumococcal Protein Vaccine Candidates: Monovalent Choline-Binding Protein A (PcpA) Vaccine and Bivalent PcpA-Pneumococcal Histidine Triad Protein D Vaccine. Vaccine (2012) 30(52):7461–8. 10.1016/j.vaccine.2012.10.076 23123106

[19] Kamtchoua T Bologa M Hopfer R Neveu D Hu B Sheng X. Safety and Immunogenicity of the Pneumococcal Pneumolysin Derivative PlyD1 in a Single-Antigen Protein Vaccine Candidate in Adults. Vaccine (2013) 31(2):327–33. 10.1016/j.vaccine.2012.11.005 23153437

[20] Odutola A Ota MO Ogundare EO Antonio M Owiafe P Worwui A. Reactogenicity, Safety and Immunogenicity of a Protein-Based Pneumococcal Vaccine in Gambian Children Aged 2–4 Years: A Phase II Randomized Study. Hum Vaccines Immunother (2016) 12(2):393–402. 10.1080/21645515.2015.1111496 PMC5049746 26618243

[21] Hammitt LL Campbell JC Borys D Weatherholtz RC Reid R Goklish N. Efficacy, Safety and Immunogenicity of a Pneumococcal Protein-Based Vaccine Co-Administered With 13-Valent Pneumococcal Conjugate Vaccine Against Acute Otitis Media in Young Children: A Phase IIb Randomized Study. Vaccine (2019) 37(51):7482–92. 10.1016/j.vaccine.2019.09.076 31629570

[22] Brooks WA Chang L-J Sheng X Hopfer R. Safety and Immunogenicity of a Trivalent Recombinant PcpA, PhtD, and PlyD1 Pneumococcal Protein Vaccine in Adults, Toddlers, and Infants: A Phase I Randomized Controlled Study. Vaccine (2015) 33(36):4610–7. 10.1016/j.vaccine.2015.06.078 26143615

[23] Entwisle C Hill S Pang Y Joachim M McIlgorm A Colaco C. Safety and Immunogenicity of a Novel Multiple Antigen Pneumococcal Vaccine in Adults: A Phase 1 Randomised Clinical Trial. Vaccine (2017) 35(51):7181–6. 10.1016/j.vaccine.2017.10.076 29132988

[24] Lu J Sun T Wang D Dong Y Xu M Hou H. Protective Immune Responses Elicited by Fusion Protein Containing PsaA and PspA Fragments. Immunol Invest (2015) 44(5):482–96. 10.3109/08820139.2015.1037956 26107747

[25] Chen A Mann B Gao G Heath R King J Maissoneuve J. Multivalent Pneumococcal Protein Vaccines Comprising Pneumolysoid With Epitopes/Fragments of CbpA and/or PspA Elicit Strong and Broad Protection. Clin Vaccine Immunol (2015) 22(10):1079–89. 10.1128/CVI.00293-15 PMC4580740 26245351

[26] Mann B Thornton J Heath R Wade KR Tweten RK Gao G. Broadly Protective Protein-Based Pneumococcal Vaccine Composed of Pneumolysin Toxoid-CbpA Peptide Recombinant Fusion Protein. J Infect Dis (2014) 209(7):1116–25. 10.1093/infdis/jit502 PMC3952665 24041791

[27] Weiser JN Ferreira DM Paton JC. Streptococcus Pneumoniae: Transmission, Colonization and Invasion. Nat Rev Microbiol (2018) 16(6):355–67. 10.1038/s41579-018-0001-8 PMC5949087 29599457

[28] Simell B Auranen K Käyhty H Goldblatt D Dagan R O’Brien KL. The Fundamental Link Between Pneumococcal Carriage and Disease. Expert Rev Vaccines (2012) 11(7):841–55. 10.1586/erv.12.53 22913260

[29] Francis JP Richmond PC Pomat WS Michael A Keno H Phuanukoonnon S. Maternal Antibodies to Pneumolysin But Not to Pneumococcal Surface Protein A Delay Early Pneumococcal Carriage in High-Risk Papua New Guinean Infants. Clin Vaccine Immunol: CVI (2009) 16(11):1633–8. 10.1128/CVI.00247-09 PMC2772384 19776196

[30] Aho C Michael A Yoannes M Greenhill A Jacoby P Reeder J. Limited Impact of Neonatal or Early Infant Schedules of 7-Valent Pneumococcal Conjugate Vaccination on Nasopharyngeal Carriage of Streptococcus Pneumoniae in Papua New Guinean Children: A Randomized Controlled Trial. Vaccine Rep (2016) 6:36–43. 10.1016/j.vacrep.2016.08.002 28580433 PMC5446595

[31] Rahman T de Gier C Orami T Seppanen EJ Granland CM Francis JP. PCV10 Elicits Protein D IgG Responses in Papua New Guinean Children But Has No Impact on NTHi Carriage in the First Two Years of Life. Vaccine (2021) 39(26):3486–92. 10.1016/j.vaccine.2021.05.022 34024658

[32] Lehmann D Kirarock W van den Biggelaar AHJ Passey M Jacoby P Saleu G. Rationale and Methods of a Randomized Controlled Trial of Immunogenicity, Safety and Impact on Carriage of Pneumococcal Conjugate and Polysaccharide Vaccines in Infants in Papua New Guinea. Pneumonia (Nathan) (2017) 9:20. 10.1186/s41479-017-0044-z 29299402 PMC5742486

[33] Satzke C Turner P Virolainen-Julkunen A Adrian PV Antonio M Hare KM. Standard Method for Detecting Upper Respiratory Carriage of Streptococcus Pneumoniae: Updated Recommendations From the World Health Organization Pneumococcal Carriage Working Group. Vaccine (2013) 32(1):165–79. 10.1016/j.vaccine.2013.08.062 24331112

[34] Pomat WS van den Biggelaar AHJ Wana S Francis JP Solomon V Greenhill AR. Safety and Immunogenicity of Pneumococcal Conjugate Vaccines in a High-Risk Population: A Randomized Controlled Trial of 10-Valent and 13-Valent Pneumococcal Conjugate Vaccine in Papua New Guinean Infants. Clin Infect Dis (2019) 68(9):1472–81. 10.1093/cid/ciy743 PMC6481999 30184183

[35] de Gier C Granland CM Pickering JL Walls T Bhuiyan M Mills N. PCV7- and PCV10-Vaccinated Otitis-Prone Children in New Zealand Have Similar Pneumococcal and Haemophilus Influenzae Densities in Their Nasopharynx and Middle Ear. Vaccines (Basel) (2019) 7(1):14. 10.3390/vaccines7010014 PMC6466140 30708945

[36] Lang ALS McNeil SA Hatchette TF Elsherif M Martin I LeBlanc JJ. Detection and Prediction of Streptococcus Pneumoniae Serotypes Directly From Nasopharyngeal Swabs Using PCR. J Med Microbiol (2015) 64(8):836–44. 10.1099/jmm.0.000097 26066632

[37] Bhuiyan MU Snelling TL West R Lang J Rahman T Granland C. The Contribution of Viruses and Bacteria to Community-Acquired Pneumonia in Vaccinated Children: A Case-Control Study. Thorax (2019) 74(3):261–9. 10.1136/thoraxjnl-2018-212096 PMC6467248 30337417

[38] de Gier C Pickering JL Richmond PC Thornton RB Kirkham LA. Duplex Quantitative PCR Assay for Detection of Haemophilus Influenzae That Distinguishes Fucose- and Protein D-Negative Strains. J Clin Microbiol (2016) 54(9):2380–3. 10.1128/JCM.00982-16 PMC5005495 27335148

[39] Wiertsema SP Corscadden KJ Mowe EN Zhang G Vijayasekaran S Coates HL. IgG Responses to Pneumococcal and Haemophilus Influenzae Protein Antigens Are Not Impaired in Children With a History of Recurrent Acute Otitis Media. PloS One (2012) 7(11):e49061. 10.1371/journal.pone.0049061 23152850 PMC3495775

[40] Thornton RB Kirkham LS Corscadden KJ Coates HL Vijayasekaran S Hillwood J. No Evidence for Impaired Humoral Immunity to Pneumococcal Proteins in Australian Aboriginal Children With Otitis Media. Int J Pediatr Otorhinolaryngol (2017) 92:119–25. 10.1016/j.ijporl.2016.11.019 28012512

[41] Francis JP Richmond PC Michael A Siba PM Jacoby P Hales BJ. A Longitudinal Study of Natural Antibody Development to Pneumococcal Surface Protein A Families 1 and 2 in Papua New Guinean Highland Children: A Cohort Study. Pneumonia (Nathan) (2016) 8:12. 10.1186/s41479-016-0014-x 28702291 PMC5471893

[42] Holmlund E Quiambao B Ollgren J Nohynek H Käyhty H. Development of Natural Antibodies to Pneumococcal Surface Protein A, Pneumococcal Surface Adhesin A and Pneumolysin in Filipino Pregnant Women and Their Infants in Relation to Pneumococcal Carriage. Vaccine (2006) 24(1):57–65. 10.1016/j.vaccine.2005.07.055 16115703

[43] Holmlund E Quiambao B Ollgren J Jaakkola T Neyt C Poolman J. Antibodies to Pneumococcal Proteins PhtD, CbpA, and LytC in Filipino Pregnant Women and Their Infants in Relation to Pneumococcal Carriage. Clin Vaccine Immunol (2009) 16(6):916–23. 10.1128/CVI.00050-09 PMC2691055 19403781

[44] Briles DE Hollingshead SK King J Swift A Braun PA Park MK. Immunization of Humans With Recombinant Pneumococcal Surface Protein A (Rpspa) Elicits Antibodies That Passively Protect Mice From Fatal Infection With Streptococcus Pneumoniae Bearing Heterologous PspA. J Infect Dis (2000) 182(6):1694–701. 10.1086/317602 11069242

[45] Hua CZ Hu WL Shang SQ Li JP Hong LQ Yan J. Serum Concentrations of Antibodies Against Outer Membrane Protein P6and T- and B-Cell Combined Antigenic Epitopes of Nontypeable Haemophilus Influenzae in Children and Adults of Different Ages. Clin Vaccine Immunol (2016) 23(2):155–61. 10.1128/CVI.00506-15 PMC4744915 26677200

[46] Thornton RB Kirkham LS Corscadden KJ Wiertsema SP Fuery A Jones BJ. Australian Aboriginal Children With Otitis Media Have Reduced Antibody Titers to Specific Nontypeable Haemophilus Influenzae Vaccine Antigens. Clin Vaccine Immunol (2017) 24(4):e00556–16. 10.1128/CVI.00556-16 PMC5382827 28151410

[47] Pichichero ME Kaur R Casey JR Sabirov A Khan MN Almudevar A. Antibody Response to Haemophilus Influenzae Outer Membrane Protein D, P6, and OMP26 After Nasopharyngeal Colonization and Acute Otitis Media in Children. Vaccine (2010) 28(44):7184–92. 10.1016/j.vaccine.2010.08.063 PMC3959881 20800701

[48] Jennewein MF Goldfarb I Dolatshahi S Cosgrove C Noelette FJ Krykbaeva M. Fc Glycan-Mediated Regulation of Placental Antibody Transfer. Cell (2019) 178(1):202–15.e14. 10.1016/j.cell.2019.05.044 31204102 PMC6741440

[49] Fu C Lu L Wu H Shaman J Cao Y Fang F. Placental Antibody Transfer Efficiency and Maternal Levels: Specific for Measles, Coxsackievirus A16, Enterovirus 71, Poliomyelitis I-III and HIV-1 Antibodies. Sci Rep (2016) 6:38874. 10.1038/srep38874 27934956 PMC5146964

[50] Novotny LA Goodman SD Bakaletz LO. Redirecting the Immune Response Towards Immunoprotective Domains of a DNABII Protein Resolves Experimental Otitis Media. NPJ Vaccines (2019) 4(1):43. 10.1038/s41541-019-0137-1 31632744 PMC6791836

[51] Papastamatiou T Routsias JG Koutsoni O Dotsika E Tsakris A Spoulou V. Evaluation of Protective Efficacy of Selected Immunodominant B-Cell Epitopes Within Virulent Surface Proteins of Streptococcus Pneumoniae. Infect Immun (2018) 86(3):e00673–17. 10.1128/IAI.00673-17 PMC5820952 29263108

