# Supplementary material for: Differences in Pneumococcal and Haemophilus influenzae Natural Antibody Development in Papua New Guinean Children in the First Year of Life
Source: Front Immunol. 2021 Aug 10;12:725244. doi: 10.3389/fimmu.2021.725244 (PMC8383109; doi:10.3389/fimmu.2021.725244)
Supplement: Supplementary Table 1 — Nontypeable Haemophilus influenzae and Streptococcus pneumoniae antigen serum IgG GMTs at each age after either PCV10 and PCV13 vaccinated groups. As we included children who had only 5 of the 6 serum samples collected in this analysis some time points do not have n=101. All values are AU/mL. [file Table_1.docx]

**Supplementary Table 1. Nontypeable *Haemophilus influenzae* and *Streptococcus pneumoniae* antigen serum IgG GMTs at each age after either PCV10 and PCV13 vaccinated groups.** As we included children who had only 5 of the 6 serum samples collected in this analysis some time points do not have n=101. All values are AU/mL.

|  | **4 months** | | | **9 months** | | | **10 months** | | | **23 months** | | | **24 months** | | |
| --- | --- | --- | --- | --- | --- | --- | --- | --- | --- | --- | --- | --- | --- | --- | --- |
|  | **PCV10**  **n=52** | **PCV13**  **n=42** | ***p*** | **PCV10**  **n=54** | **PCV13**  **n=44** | ***p*** | **PCV10**  **n=55** | **PCV13**  **n=44** | ***p*** | **PCV10**  **n=48** | **PCV13**  **n=45** | ***p*** | **PCV10**  **n=52** | **PCV13 n=43** | ***p*** |
| **Nontypeable *Haemophilus influenzae* antigens** | | | | | | | | | | | | | | | |
| **P4** | 58614 | 41591 | *0.650* | 46774 | 37239 | *0.420* | 58614 | 36559 | *0.117* | 72778 | 60256 | *0.293* | 69502 | 59293 | *0.410* |
| **P6** | 317687 | 257632 | *0.404* | 389942 | 339625 | *0.571* | 492040 | 355631 | *0.198* | 895365 | 727780 | *0.263* | 818465 | 746449 | *0.604* |
| **OMP26** | 80910 | 57544 | *0.243* | 113763 | 129718 | *0.650* | 191426 | 156675 | *0.454* | 328852 | 295121 | *0.573* | 322849 | 341979 | *0.753* |
| **rsPilA** | 8375 | 6561 | *0.378* | 7047 | 8035 | *0.533* | 9162 | 8750 | *0.802* | 13002 | 13366 | *0.864* | 14191 | 13996 | *0.926* |
| **ChimV4** | 25468 | 19187 | *0.375* | 14555 | 19999 | *0.275* | 19588 | 21429 | *0.746* | 38994 | 33420 | *0.470* | 38019 | 31261 | *0.309* |
| ***Streptococcus pneumoniae* antigens** | | | | | | | | | | | | | | | |
| **PspA1** | 31915 | 34119 | *0.740* | 10914 | 12972 | *0.580* | 15959 | 16406 | *0.939* | 69984 | 64863 | *0.802* | 71285 | 56885 | *0.460* |
| **PspA2** | 102329 | 125026 | *0.367* | 39355 | 64714 | *0.199* | 55463 | 87700 | *0.225* | 372392 | 358096 | *0.909* | 393550 | 350752 | *0.744* |
| **CbpA** | 276694 | 293089 | *0.813* | 717794 | 517607 | *0.309* | 1042317 | 578096 | *0.048* | 2897344 | 2716439 | *0.693* | 2857591 | 2606154 | *0.564* |
| **Ply** | 144212 | 169044 | *0.495* | 154882 | 224388 | *0.298* | 244906 | 257632 | *0.882* | 1749847 | 1682674 | *0.257* | 1428894 | 1644372 | *0.464* |

PCV10, 10-valent pneumococcal conjugate vaccine; PCV13, 13-valent pneumococcal conjugate vaccine; PD, Protein D; P4, Protein 4; P6, outer membrane protein 6, OMP26, outer membrane protein 26; rsPilA, recombinant soluble pilus A protein; ChimV4, chimeric vaccine antigen 4 (rsPilA and P5); PspA1, pneumococcal surface protein A family 1; PspA2, pneumococcal surface protein A family 2; CbpA, choline-binding protein A; Ply, non-toxic derivatives of pneumolysin . *p*-values are in italics and calculated by t-test comparing log-transformed titres between groups at each age.
