# Supplementary material for: Differences in Pneumococcal and Haemophilus influenzae Natural Antibody Development in Papua New Guinean Children in the First Year of Life
Source: Front Immunol. 2021 Aug 10;12:725244. doi: 10.3389/fimmu.2021.725244 (PMC8383109; doi:10.3389/fimmu.2021.725244)
Supplement: Supplementary Table 2 — Nontypeable Haemophilus influenzae and Streptococcus pneumoniae antigen serum IgG GMTs compared between PPV23 recipients and non-recipients for those who received PCV10 or PCV13. As we included children who had only 5 of the 6 serum samples collected in this analysis some time points do not have n=101. All values are AU/mL. [file Table_2.docx]

**Supplementary Table 2. Nontypeable *Haemophilus influenzae* and *Streptococcus pneumoniae* antigen serum IgG GMTs compared between PPV23 recipients and non-recipients for those who received PCV10 or PCV13.** As we included children who had only 5 of the 6 serum samples collected in this analysis some time points do not have n=101. All values are AU/mL.

|  | | **10 months** | | | **23 months** | | | **24 months** | | |
| --- | --- | --- | --- | --- | --- | --- | --- | --- | --- | --- |
|  |  | **PPV23+** | **PPV23-** | ***p*** | **PPV23+** | **PPV23-** | ***p*** | **PPV23+** | **PPV23-** | ***p*** |
| **PCV10 n=** | | **33** | **22** |  | **29** | **19** |  | **32** | **20** |  |
| **PCV13 n=** | | **25** | **19** |  | **26** | **19** |  | **26** | **17** |  |
| **Nontypeable *Haemophilus influenzae* antigens** | | | | | | | | | | |
| **P4** | **PCV10** | 57810 | 59841 | *0.938* | 82414 | 60256 | *0.213* | 809096 | 665273 | *0.015* |
|  | **PCV13** | 41115 | 31405 | *0.518* | 56885 | 65163 | *0.612* | 350752 | 282488 | *0.294* |
| **P6** | **PCV10** | 435512 | 591562 | *0.358* | 920450 | 859014 | *0.781* | 377572 | 293765 | *0.664* |
|  | **PCV13** | 433511 | 274158 | *0.250* | 660693 | 827942 | *0.440* | 16218 | 11508 | *0.501* |
| **OMP26** | **PCV10** | 184927 | 201837 | *0.806* | 328095 | 330370 | *0.982* | 15959 | 11455 | *0.381* |
|  | **PCV13** | 204644 | 110154 | *0.146* | 301995 | 287078 | *0.872* | 45290 | 28642 | *0.397* |
| **rsPilA** | **PCV10** | 9441 | 8770 | *0.775* | 14223 | 11350 | *0.303* | 35237 | 26002 | *0.075* |
|  | **PCV13** | 8630 | 8913 | *0.904* | 13836 | 12735 | *0.708* | 809096 | 665273 | *0.214* |
| **ChimV4** | **PCV10** | 19632 | 19543 | *0.989* | 41591 | 35400 | *0.557* | 350752 | 282488 | *0.060* |
|  | **PCV13** | 18578 | 25645 | *0.509* | 33806 | 32885 | *0.939* | 377572 | 293765 | *0.337* |
| ***Streptococcus pneumoniae* antigens** | | | | | | | | | | |
| **PspA1** | **PCV10** | 17824 | 13552 | *0.566* | 57280 | 94842 | *0.271* | 68549 | 75858 | *0.829* |
|  | **PCV13** | 18197 | 14256 | *0.613* | 54702 | 81846 | *0.343* | 54702 | 60534 | *0.800* |
| **PspA2** | **PCV10** | 43652 | 79616 | *0.248* | 375837 | 366438 | *0.962* | 401791 | 379315 | *0.909* |
|  | **PCV13** | 94624 | 79250 | *0.753* | 374111 | 337287 | *0.839* | 393550 | 293765 | *0.569* |
| **CbpA** | **PCV10** | 941890 | 1213389 | *0.458* | 2760578 | 3118890 | *0.571* | 2691535 | 3155005 | *0.506* |
|  | **PCV13** | 706318 | 444631 | *0.376* | 2594179 | 2884032 | *0.688* | 2710192 | 2454709 | *0.661* |
| **Ply** | **PCV10** | 289068 | 190985 | *0.356* | 1534617 | 1166810 | *0.391* | 1479108 | 1352073 | *0.753* |
|  | **PCV13** | 252930 | 264850 | *0.938* | 1438799 | 2254239 | *0.100* | 1573983 | 1757924 | *0.675* |

PPV23+, recipients of the 23-valent pneumococcal polysaccharide vaccine (PPV23) at 9 months of age; PPV23-, those who did not receive PPV23 at 9 months of age; PD, Protein D; P4, Protein 4; P6, outer membrane protein 6, OMP26, outer membrane protein 26; rsPilA, recombinant soluble pilus A protein; ChimV4, chimeric vaccine antigen 4 (rsPilA and P5); PspA1, pneumococcal surface protein A family 1; PspA2, pneumococcal surface protein A family 2; CbpA, choline-binding protein A; Ply, non-toxic derivatives of pneumolysin *p*-values are in italics and calculated by t-test comparing log-transformed tires between groups at each age.
