# Supplementary material for: Differences in Pneumococcal and Haemophilus influenzae Natural Antibody Development in Papua New Guinean Children in the First Year of Life
Source: Front Immunol. 2021 Aug 10;12:725244. doi: 10.3389/fimmu.2021.725244 (PMC8383109; doi:10.3389/fimmu.2021.725244)
Supplement: Supplementary Table 3 — S. pneumoniae density correlated with S. pneumoniae protein-specific IgG titre at different age time points. As we included children who had only 5 of the 6 serum samples collected in this analysis, time points do not have n=101. [file Table_3.docx]

**Supplementary Table 3: *S. pneumoniae* density correlated with *S. pneumoniae* protein-specific IgG titre at different age time points.** As we included children who had only 5 of the 6 serum samples collected in this analysis, time points do not have n=101.

|  | | **PspA1** | | **PspA2** | | **CbpA** | | **Ply** | |
| --- | --- | --- | --- | --- | --- | --- | --- | --- | --- |
| **Age** | **N** | **R-squared** | **p-value** | **R-squared** | **p-value** | **R-squared** | **p-value** | **R-squared** | **p-value** |
| **1 month** | 89 | 0.004 | 0.575 | 0.007 | 0.445 | 1.03 x10^-5^ | 0.976 | 0.004 | 0.568 |
| **PCV10 or PCV13 given at 1, 2, and 3 months of age** | | | | | | | | | |
| **4 months** | 94 | 0.002 | 0.655 | 0.000 | 0.876 | 0.002 | 0.700 | 0.001 | 0.734 |
| **9 months** | 98 | 0.001 | 0.735 | 0.027 | 0.104 | 0.011 | 0.312 | 0.001 | 0.809 |
| **~Half from each cohort received PPV23 at 9 months of age** | | | | | | | | | |
| **10 months** | 99 | 0.003 | 0.569 | 0.003 | 0.569 | 0.000 | 0.880 | 0.012 | 0.285 |
| **23 months** | 93 | 0.000 | 0.881 | 0.000 | 0.881 | 0.001 | 0.795 | 0.015 | 0.245 |
| **All children received 1/5^th^ dose of PPV23 at 23 months of age** | | | | | | | | | |
| **24 months** | 95 | 0.005 | 0.484 | 0.005 | 0.484 | 0.016 | 0.228 | 0.001 | 0.836 |
