# Supplementary material for: Differences in Pneumococcal and Haemophilus influenzae Natural Antibody Development in Papua New Guinean Children in the First Year of Life
Source: Front Immunol. 2021 Aug 10;12:725244. doi: 10.3389/fimmu.2021.725244 (PMC8383109; doi:10.3389/fimmu.2021.725244)
Supplement: Supplementary Table 4 — NTHi density in swabs correlated with NTHi antigen titres. As we included children who had only 5 of the 6 serum samples collected in this analysis, time points do not have n=101. [file Table_4.docx]

**Supplementary Table 4: NTHi density in swabs correlated with NTHi antigen titres.** As we included children who had only 5 of the 6 serum samples collected in this analysis, time points do not have n=101.

|  |  | **P4** | | **P6** | | **OMP26** | | **rsPilA** | | **ChimV4** | |
| --- | --- | --- | --- | --- | --- | --- | --- | --- | --- | --- | --- |
| **Age** | **N** | **R-squared** | **p-value** | **R-squared** | **p-value** | **R-squared** | **p-value** | **R-squared** | **p-value** | **R-squared** | **p-value** |
| **1 month** | 89 | 4.02 x10^-5^ | 0.953 | 0.002 | 0.691 | 0.008 | 0.407 | 0.009 | 0.377 | 0.006 | 0.480 |
| **PCV10 or PCV13 given at 1, 2, and 3 months of age** | | | | | | | | | | | |
| **4 months** | 94 | 0.001 | 0.814 | 9.26 x10^-5^ | 0.927 | 6.38 x10^-6^ | 0.981 | 0.003 | 0.604 | 0.001 | 0.840 |
| **9 months** | 98 | 0.020 | 0.165 | 0.013 | 0.263 | 6.63x10^-5^ | 0.937 | 0.001 | 0.720 | 0.032 | 0.077 |
| **~Half from each cohort received PPV23 at 9 months of age** | | | | | | | | | | | |
| **10 months** | 99 | 0.003 | 0.598 | 0.018 | 0.186 | 0.005 | 0.463 | 0.005 | 0.397 | 8.86 x10^-6^ | 0.977 |
| **23 months** | 93 | 2.71 x10^-5^ | 0.961 | 4.69 x10^-6^ | 0.984 | 0.016 | 0.235 | 0.001 | 0.878 | 0.020 | 0.173 |
| **All children received 1/5^th^ dose of PPV23 at 23 months of age** | | | | | | | | | | | |
| **24 months** | 95 | 0.003 | 0.598 | 0.001 | 0.870 | 0.001 | 0.742 | 0.015 | 0.239 | 0.003 | 0.625 |
