# Supplementary figures and images for: Differences in Pneumococcal and Haemophilus influenzae Natural Antibody Development in Papua New Guinean Children in the First Year of Life
Source: Front Immunol. 2021 Aug 10;12:725244. doi: 10.3389/fimmu.2021.725244 (PMC8383109; doi:10.3389/fimmu.2021.725244)

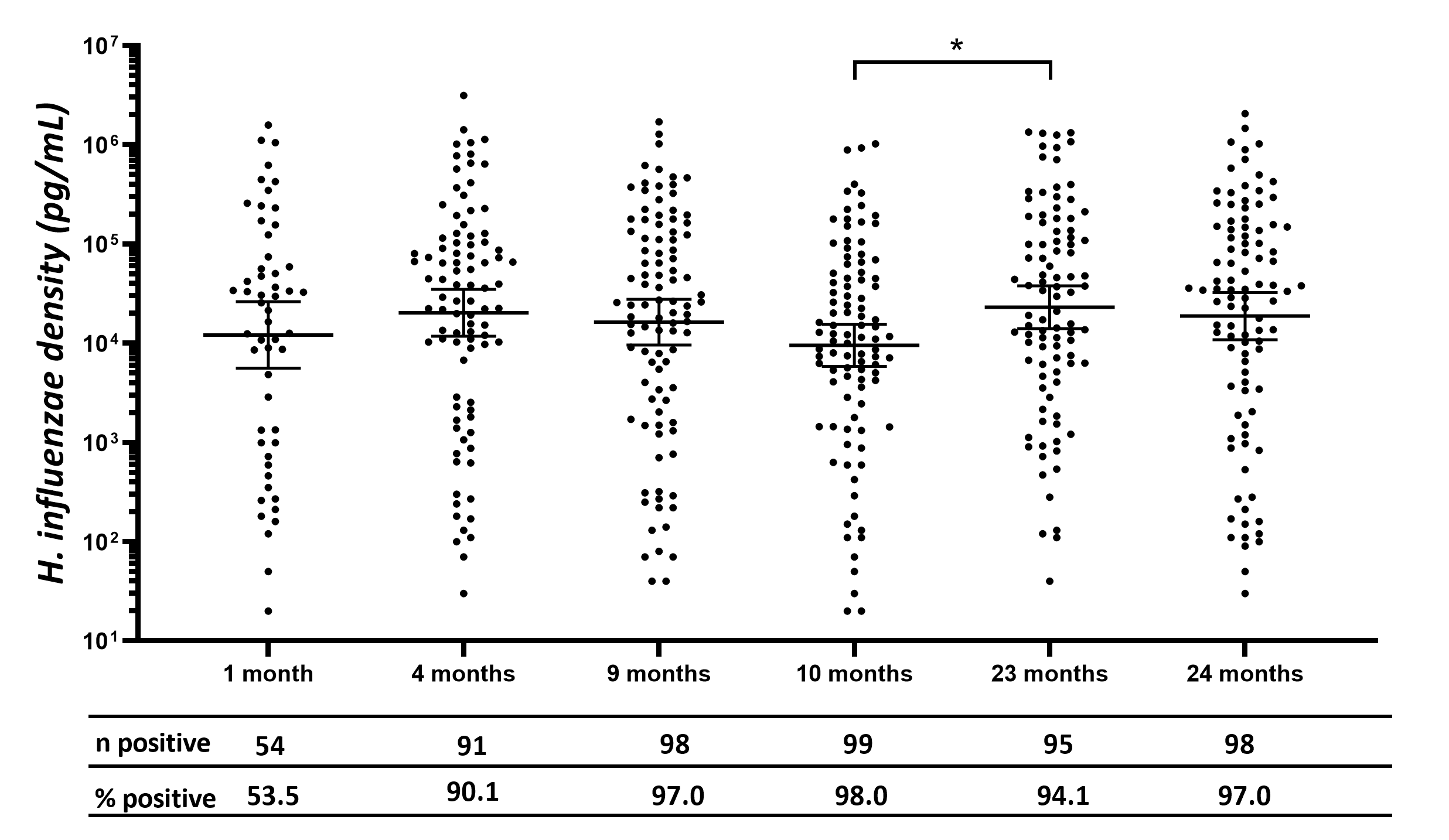

Supplement: Supplementary Figure 1 — S. pneumoniae density in children carrying S. pneumoniae up to 2 years of age. S. pneumoniae nasopharyngeal density in swabs from children taken at 1, 4, 9, 10, 23 and 24 months of age. Data are presented for children that were colonised with S. pneumoniae as determined by qPCR. Each point represents an individual child, and the horizontal bars depict the median geometric mean density of DNA concentration in µg/mL in qPCR-positive samples. The number and percentages of children who were positive for S. pneumoniae carriage (S. pneumoniae DNA detected above the assay limit of detection) is outlined underneath the graph. *p-value < 0.05. [file Image_1.tif]

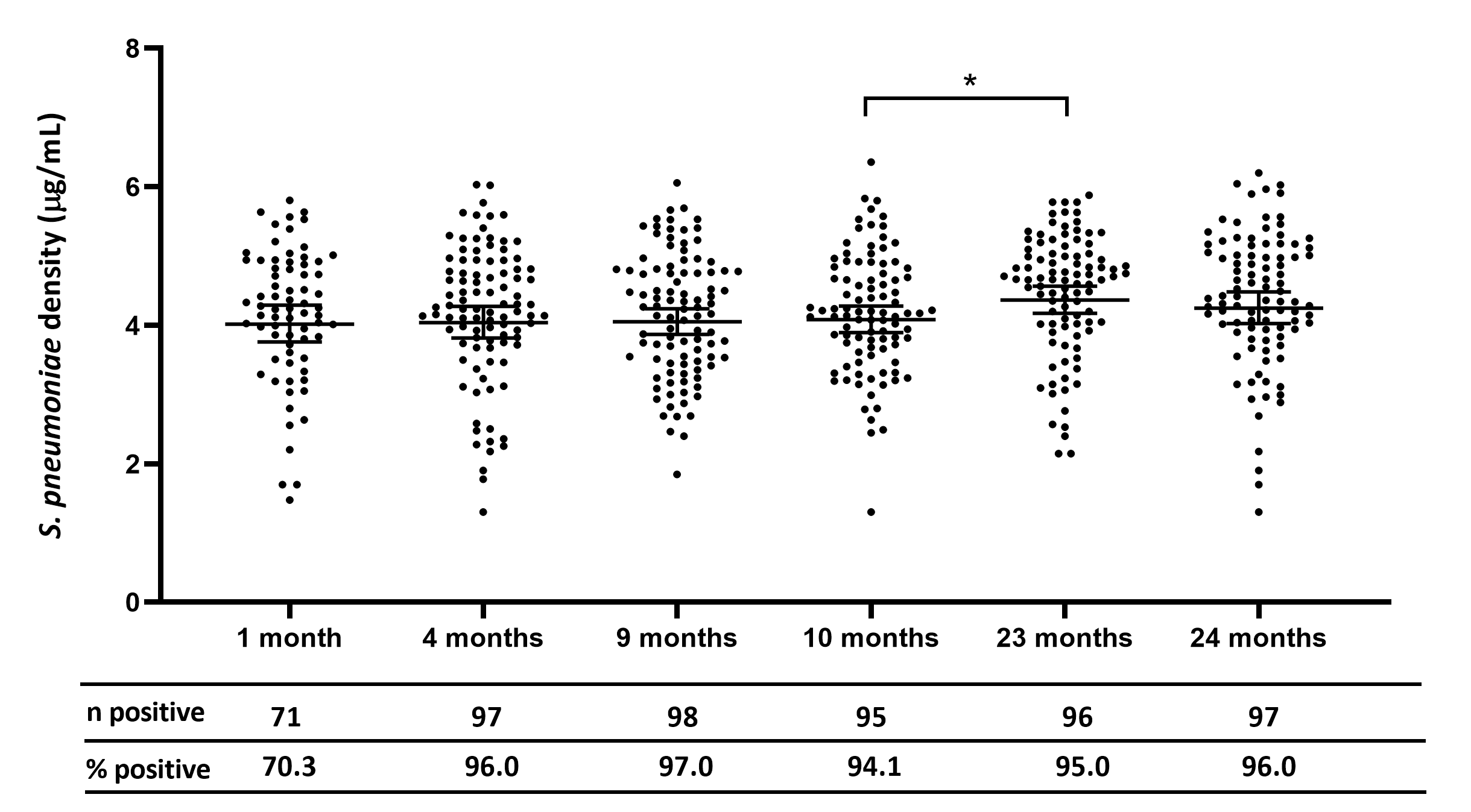

Supplement: Supplementary Figure 2 — NTHi density in children carrying NTHi up to 2 years of age. NTHi nasopharyngeal density in swabs from children taken at 1, 4, 9, 10, 23 and 24 months of age. Data are presented for children that were colonised with NTHi as determined by qPCR. Each point represents an individual child, and the horizontal bars depict the median geometric mean density of DNA concentration in µg/mL in qPCR-positive samples. The number and percentages of children who were positive for NTHi carriage (NTHi DNA detected above the assay limit of detection) is outlined underneath the graph. *p-value < 0.05. [file Image_2.tif]
